# Chagas disease mortality in Brazil: A Bayesian analysis of age-period-cohort effects and forecasts for two decades

**DOI:** 10.1371/journal.pntd.0006798

**Published:** 2018-09-28

**Authors:** Taynãna César Simões, Laiane Félix Borges, Auzenda Conceição Parreira de Assis, Maria Vitórias Silva, Juliano dos Santos, Karina Cardoso Meira

**Affiliations:** 1 Clinical Research and Public Policy in Infectious and Parasitic Diseases Group, René Rachou Institute, Oswaldo Cruz Foundation, Belo Horizonte, Minas Gerais, Brazil; 2 School of Health, Federal University of Rio Grande do Norte, Natal, Rio Grande do Norte, Brazil; 3 Ministry of Health of Brazil, São Paulo State Nucleus, São Paulo, São Paulo, Brazil; Universidad de Buenos Aires, ARGENTINA

## Abstract

**Background:**

Chagas disease (CD) is a neglected chronic parasitic infection and a public health problem that is preventable, and has serious complications. In this study, the effects of age, period and birth cohort (APC Effects) on the evolution of the mortality of that disease in Brazil, from 1980–2014, according to sex and geographic region of the country, were analyzed. Mortality forecasts from the years 2015 to 2034 were estimated.

**Methods:**

This is an ecological cross-sectional study in which death records and population data were extracted from the DATASUS (Department of Information Technology of the National Health System) website, in age groups from 20–24 years of age to 80 years and over, from 1980 to 2014. The rates were standardized according to age and sex distributions using the direct method. The APC models were estimated using the Bayesian approach, and the INLA (Integrated Nested Laplace Approximations) method was used for parameter inference. Super dispersion of the data was considered, and we included unstructured random terms in the models.

**Results:**

During the analyzed period, there were 178,823 deaths in Brazil (3.85 annual deaths per 100,000 inhabitants). It was found that temporal effects on CD mortality varied by sex and region. In general, there was an increase in mortality rates up to 30 years of age, and the mortality rates were higher between 50 and 64 years of age. On average, men died five years younger than women. Mortality rates were highest in the Central West and Southeast regions. The Central West, Southeast and Southern regions had a reduction over time in the rate of CD deaths between 2000 and 2014. The mortality rate in the Northeast was not statistically different in any period analyzed, while the North had tendency to increase; however, a significant risk increase was only observed between 1995 and 1999. The rate of mortality was high in older birth cohorts. The overall prediction for the next two decades showed a progressive decline in CD mortality, which will be highest among the young. The expected average reduction was 76.1% compared to the last observed period (2010–2014) and the last predicted (2030–2034) period. The average reduction ranged from 86% in the 20–24 age group to 50% in the 80 and over age group.

**Conclusions:**

In the present study, a higher death rate was observed for ages above 30 years, especially 50 to 64 years, and in the older birth cohorts. We believe these results can be related to period effects of vector control actions and preventive and care measures by the health system of Brazil, in addition to demographic changes in the period. The differences among the regions reflect socioeconomic inequities and access to the healthcare systems in the Brazilian population.

## Author summary

Chagas disease (CD) is a neglected chronic parasitic infection of great interest in public health, because it is preventable and has serious complications. In Brazil, despite the success obtained by controlling transmission by the *T*.*infestans* vector and by transfusion screening, the disease still affects more than 4 million people, with 6000 annual deaths. Studies that evaluate mortality from CD are an important indicator of the impact of the disease. However, studies in Brazil have not evaluated the effects of age, period and birth cohort (APC effects) separately to understand the importance and particularity of aspects related to the evolution of CD mortality over time. This study estimated the APC effects by sex and geographic region of Brazil in the period from 1980–2014 and estimated the mortality rates for two more decades (2015–2034). Using Bayesian hierarchical modeling, significant differences in temporal effects were found. The main findings were as follows: a) a progressive increase in the rate of CD death with increasing age, up to 30 years, with the highest mortality between 50 and 64 years; b) a generational effect, with a lower rate of death for younger cohorts; c) a reduction in the rate of CD death for the Central West, South and Southeast regions no statistical effect in the Northeast region, and increased risk in the North region since 2000; and d) a reduction in the mortality rates for the next 20 years, with rates being highest among the youngest age group. These results suggest the importance of the period effect on Chagas disease prevention and control measures, in addition to the expansion of health services access and the migration from endemic areas to non-endemic areas that has reduced population exposure to *T*.*cruzi* infection, especially in birth cohorts after the 1960s. In addition, differences among the regions of the country reflect the association of disease mortality with socioeconomic status, as well as inequities in access to healthcare by the Brazilian population.

## Introduction

Chagas disease (CD) is a chronic parasitic infection caused by Trypanosoma cruzi. *T*. *cruzi* is a complex zoonotic parasite that has numerous vertebrate reservoirs and triatomine insects as vectors. It is considered a neglected tropical disease. CD has higher prevalence and mortality in populations of high social vulnerability, generating a significant socioeconomic impact due to early mortality, loss of years of life due to disability and high costs due to medical care and social security [1–5].

It is estimated that between 6 and 7 million people are infected with CD worldwide. Of these, 5 million are in Latin America, where more than 70 million people are at risk of infection and 12,000 die annually [4],[6]. However, although CD is more prevalent in this region, surveillance and control actions in Europe and North America are also important due to migratory processes that increase transmission risk, blood transfusion, organ donation and congenital transmission. In this context, of 1,678,711 Latin American immigrants received in Spain in 2007, 5.2% were potentially infected with *T*. *cruzi* and 24–92 newborns delivered by infected mothers may have been congenitally infected [7],[8].

In Brazil, it is estimated that from 1 to 4.6 million people are currently infected with this parasite, and despite prevention and control advances in the last 40 years, there are still approximately 6,000 deaths per year [5],[9–11]. In Brazil, CD was the fourth leading cause of death among infectious and parasitic diseases in the period from 1999 to 2007 [12], and in the period from 2000 to 2011, it represented 76.7% of all deaths due to neglected tropical diseases, with a mortality rate of 3.37 deaths / 100,000 inhabitants [13]. In this context, it is necessary to monitor the evolution of mortality by CD, in order to support the evaluation and planning of health policies.

In 2014, Martins-Melo and colleagues estimated an average annual mortality rate per DC of 3.37/100 thousand inhabitants in the country [14]. A downward trend in mortality from this disease has been observed in the country in the last decades; the decrease is heterogeneous among its five geographic regions, which are markedly different from each other in relation to demographic and socioeconomic characteristics [10],[12],[15–20].

Existing studies have shown that the highest mortality rates occur in the Central West, Southeast and South regions, areas of high endemicity in the past and that received a high migratory flow of individuals from endemic regions in the 1950s-60s. On the other hand, the North and Northeast regions, which have the lowest rates of socioeconomic development in the country, show the lowest mortality rates due to CD, with upward or stationary trends, depending on the time period analyzed. These studies also point out that the highest mortality rates occur in men above 60 years of age and include cardiac involvement as the main complication associated with the disease [10],[12],[15–20].

Although the aforementioned studies have contributed to the surveillance and knowledge of the epidemiological profile of the mortality burden due to CD in the last 15 years, most studies do not consider the possible influence of birth cohorts on the temporal change in mortality from that CD. Moreover, the few studies that evaluated birth cohort effects in this context has analyzed the effects only in an exploratory way, without considering probabilistic models [20],[21].

Age, period, and birth cohort are components (APC effects) that can influence, separately or together, the time trend of an event. In the health context, the effect of age may reflect physiological changes that occur in the individual throughout life, increasing or reducing the risk of illness and/or death. The period effect is related to structural changes affecting all age groups simultaneously, such as innovations in diagnostic and therapeutic techniques, expansion of access to health services, and improvements in the certification of deaths. The birth cohort represents the interaction between the effects of age and period, representing the influence of factors of exposure throughout life and possible determinants of disease and/or mortality [22–26].

Because Chagas disease in its chronic phase is a long latency disease, responsible for more than 90% of mortalities, it is believed that different birth cohorts will have differences in exposure to risk and protection factors for this disease. Since *T*. *cruzi* infection (vector, transfusion, oral and congenital) is the main risk factor for this disease and Brazil has participated in international agreements for the prevention and control of CD, it is possible to affirm that younger cohorts have a lower risk of death from CD due to structural changes in control and prevention, as well as access to health services in the country over time. Thus, the purpose of the present study is to analyze the temporal evolution of mortality due to CD in Brazil from 1980–2014, according to the effects of age, period and birth cohort, by sex and region of the country; we also estimated these rates for the next 20 years (2015–2034).

## Methods

### Ethics statement

This study analyzes secondary data, available for free download on the DATASUS—Departamento de Informática do SUS (Department of Information Technology of the National Health System) website from the Ministry of Health of Brazil. On this website, the city level is the smallest geographic unit available, so it is impossible to identify the individual. Thus, it was not necessary to submit the study to the local ethics committee because all data analyzed were anonymized.

### Study design and data source

This is an ecological study of the temporal trend of CD mortality rates in Brazil. Brazil has a population of approximately 207.7 million inhabitants. The country is divided into 26 States and one Federal government. The Federation is further grouped into five major regions (North, Northeast, Southeast, South and Central West) with different geographic, economic and cultural characteristics. The best human development indexes are observed in the South. The Southeast, the most populous region, stands out for its labor market. The Central West, although it includes the capital of the country, has an economy focused on agriculture and livestock. The Northeast region has the lowest human development rates. And finally, The North occupies second place for the worst development rates in the country and is characterized by its low population density due to the Amazon forest.

The annual records of deaths and population data from 1980 to 2014, according to sex and Brazilian geographic region, specific for the age groups 0–4 years to 80 years and over, were extracted from DATASUS, a national electronic database from the Ministry of Health of Brazil. The population data, provided by IBGE (Brazilian Institute of Geography and Statistics), could also be downloaded from the DATASUS website.

The last two years available in the system were discarded, because we wanted five-year ranges. The underlying cause of death selected in the Mortality Information System (SIM/DATASUS) was Trypanosomiasis—codes 086 (ICD-9) and B57 (ICD-10). Death certificates contain demographic (age, gender, education, race, marital status, municipality of residence and municipality of occurrence of death) and clinical information (underlying and associated causes of death). It is the physicians' responsibility to complete the death certificates. Until 1995, reference codes were based on the International Classification of Diseases (ICD) in its 9th revision, and after 1996, they were based on the 10th revision of the ICD [27].

The correction process was used because of the great disparity in information quality among the Brazilian geographic regions, and because during the period under study, there were important improvements in death registry quality in Brazil due to the expansion of health services with the implementation of the Unified Health System (SUS) [28]. In addition, Holford (1991) affirms that improvements in death certificates may have a period effect and so, in order not to draw erroneous conclusions regarding the evolution Chagas disease mortality, we have chosen to correct death records [23]. Due to differences in the quality of information included in death records among the geographic regions of the country [29], deaths were corrected in three stages: (1) we performed a proportional redistribution of records classified with age and/or sex ignored among the deaths classified as having an underlying cause of death due to Chagas disease. (2) The treatment of ill-defined deaths was performed using codes 780–799 in CID 9 and codes R00-R99 in CID 10.

The coverage degree of the external causes of death was close to 100%. Thus, these reports were excluded, considering only "natural" causes of death (natural causes of death = (total of all causes of death) minus (ill-defined causes of death + external causes)). The proportional redistribution of deaths by year and age group was also performed using the following CD correction: underlying CD deaths + (underlying CD deaths / "natural" causes of death). (3) The deaths obtained in steps 1 and 2 were added to the death records initially classified as CD death, per year and age group.

Subsequently, mortality rates per 100,000 inhabitants were calculated according to sex, age group and geographic region and were standardized using the direct method, using the Brazilian population of the 2010 Census as the standard. The average five-year rates per 100,000 inhabitants, crude and standardized, were calculated, as well as the rates for the whole observed period. We decided to use the age groups and annual periods grouped in intervals of five years, a strategy commonly adopted in APC modeling. We also chose to work with age groups older than 20–24 years, considering the excess of zeros in the age groups younger than 20 years. Thus, we considered 13 age groups, 7 periods, and 19 birth cohorts (1900 to 1990).

### Modeling procedure

The classical proposals that model the interest rate as a result of a linear predictor formed by the sum of the temporal components (age, period and cohort) are problematic due to the linear relationship among those three components, making the complete models unidentifiable. Several proposals have been developed in order to solve this problem, but there is still no consensus or definitive solution in the literature [22],[30–34]. In recent years, the APC models under the Bayesian approach have been used. Such models assume that time effects that are close are similar, attributing priors probability distributions, which softens the effects of age, period, and cohort. In addition, hierarchical Bayesian models allow the incorporation of additional random effects in a less complex way, with and without temporal and/or spatial structure [35],[36].

In a Bayesian context, the APC model is a hierarchical model that incorporates uncertainty about hyper parameters and avoids difficulties arising from the identifiability problem by the application of mildly informative prior distributions. In addition, the Bayesian model is very popular for projection rates because it does not rely on strong parametric assumptions for future values of period and cohort effects. Smoothing priors represents an attractive alternative to maximum likelihood (ML) based approaches when age groups and periods are given for the same time-interval widths and avoids artifacts such as artificial cyclical patterns [35].

We have age-specific rates in one set of rates for each stratum, sex and geographical region. Each set of rates could be analyzed separately by means of a classical APC model. However, because of similar relevant risk factors, it was beneficial to analyze all sets of rates jointly, treating some sets of time effects as common across strata. The major advantage of using the classical approach in the present study is due to the possibility of statistically testing the difference in effects estimated for the factor levels, such as sex and region, so we would not need to assume a priori that there is a significant difference among these levels, as in the classical method, making it impossible to perform a formal test. Multivariate APC models share sets of time effects (i.e., age effects), while the remaining parameters can be different. The differences in the stratum-specific time effects in multivariate APC models are identifiable and can be interpreted as log relative risks so that the identifiability problem for univariate APC models is avoided [35],[36].

As a modeling strategy, first, a univariate Bayesian APC model was adjusted to estimate global temporal effects on the risk of CD death in the country [37]. The ratio between the category estimates of the time variables provides the measure of association Rate Ratio (RRR), which quantifies the relative difference between the rate of two categories, for example, ages *i* and *i* + 1. If there is a protective effect, we refer to it as 0 < RRR < 1, and if there is a risk effect, it is referred to as RRR > 1. Subsequently, multivariate models were adjusted, where *y*_*ijg*_ and *n*_*ijg*_ were the number of deaths and number of people at risk in the age group *i* = 1,…,13, period *j* = 1,…,7, and sex category or region *g*, respectively [35]. Assuming constant period effects between sex/region categories, the following model was used as an example:

$$\begin{array}{c} y_{ijg}∼Poisson\;\left(n_{ijg}exp(\varepsilon_{ijg})\right)\\ \varepsilon_{ijg}=\mu_{g}+\alpha_{ig}+\beta_{j}+\gamma_{kg}+z_{ijg}+ln(n_{ijg}) \end{array},$$

where, *μ*_*g*_ is the global mean specific for sex/region *g*, *α*_*ig*_ is the effect of age group *i* and sex / region *g*, *β*_*j*_ is the global period effect, *γ*_*kg*_ is the effect of the birth cohort *k* and sex / region *g*, *n*_*ij*_ is the number of people at risk at age *i*, period *j*, and sex / region *g*, and *z*_*ij*_~*N*(0,*δ*^−1^) are the random effects to adjust for over dispersion (extra variability) [37]. The models proposed in this work are variations of the model presented above and are listed in Table 1.

**Table 1:** Proposed multivariate models considering temporal effects, overdispersion random effects, and heterogeneity by sex (or geographic region).

APC Models	Linear Predictor
Model 1a (1b)	Joint effects for men and women (or geographic regions)
Model 2a (2b)	Age-specific effects by sex (or geographic regions)
Model 3a (3b)	Period-specific effects by sex (or geographic regions)
Model 4a (4b)	Cohort-specific effects by sex (or geographic regions)
Model 5a (5b)	Age-specific and period-specific effects by sex (or geographic regions)
Model 6a (6b)	Age-specific and cohort-specific effects by sex (or geographic regions)
Model 7a (7b)	Period-specific and cohort-specific effects by sex (or geographic regions)

To ensure the intercept identifiability of *μ*, we have the restriction $\sum_{i=1}^{I}\alpha_{i}=\sum_{j=1}^{J}\beta_{j}=\sum_{k=1}^{K}\gamma_{k}=0$ for each sex/region category. However, due to the linear relationship between the temporal terms, *K* = *I* − *i* + *j*, the effects of age, period and cohort are still not identifiable. Smoothing Independent Gaussian priors were used for the main effects and a uniform priori attributed to *μ*. The nonidentifiability of the latent parameters remains, but it does not require additional constraints as in the classical models [22],[30–33].

The temporal effects received a first order random-walk (RW1) as priors, which penalize deviations from a model where all parameters are constant (*α*_1_ = ⋯ = *α*_*I*_, *i* = 2,…,*I*): *RW*1: *α*_*i*_~*N*(*α*_*i*−1_,*κ*^−1^), where *κ*,*λ* and *ν* are the smoothing parameters (precision) for the effects of age, period and cohort, respectively. In this study, noninformative priors were assigned to the precision parameters (G(1;0,00005)), and independent uniform priors were assigned to *α*_1_ and *α*_2_. These values choices are commonly used in the ecological regression literature.

For the parameter inference, the INLA (Integrated Nested Laplace Approximations) method, an alternative to MCMC (Monte Carlo via Markov Chains), was used in latent Gaussian models [38]. Unstructured random terms were tested in order to consider the overdispersion. The comparison among the models was done by the Information Deviance Criterion (DIC) and the log-score. Both are negatively oriented, in the sense that the smaller the value are, the better the fitted model [39],[40].

The Bayesian approach facilitates predictions because strong parametric assumptions are not necessary. Given the samples of the posterior distribution of the fitted model for the whole country, predictions of Chagas death rates, by age groups, were estimated up to 2034 [41]. The analyses were performed in statistical software R, using the inla and bapc packages [42].

## Results

### Describing the data

In Brazil, from 1980 to 2014, in individuals aged 0–80 and over, 178,823 deaths were classified with Chagas disease as the basic cause. The average of crude mortality rates was 2.70 deaths/100,000 inhabitants, and the average of standardized mortality rates was 3.85 deaths/100,000 inhabitants over the whole period. After correcting by ill-defined causes, with sex and age ignored, there was a 27% increase in the number of deaths due to CD (227,094 deaths), corresponding to 4.28 deaths/100,000 inhabitants (average of crude mortality rates), and an average of standardized mortality rates of 4.85 deaths/100,000 inhabitants.

**Fig 1:**
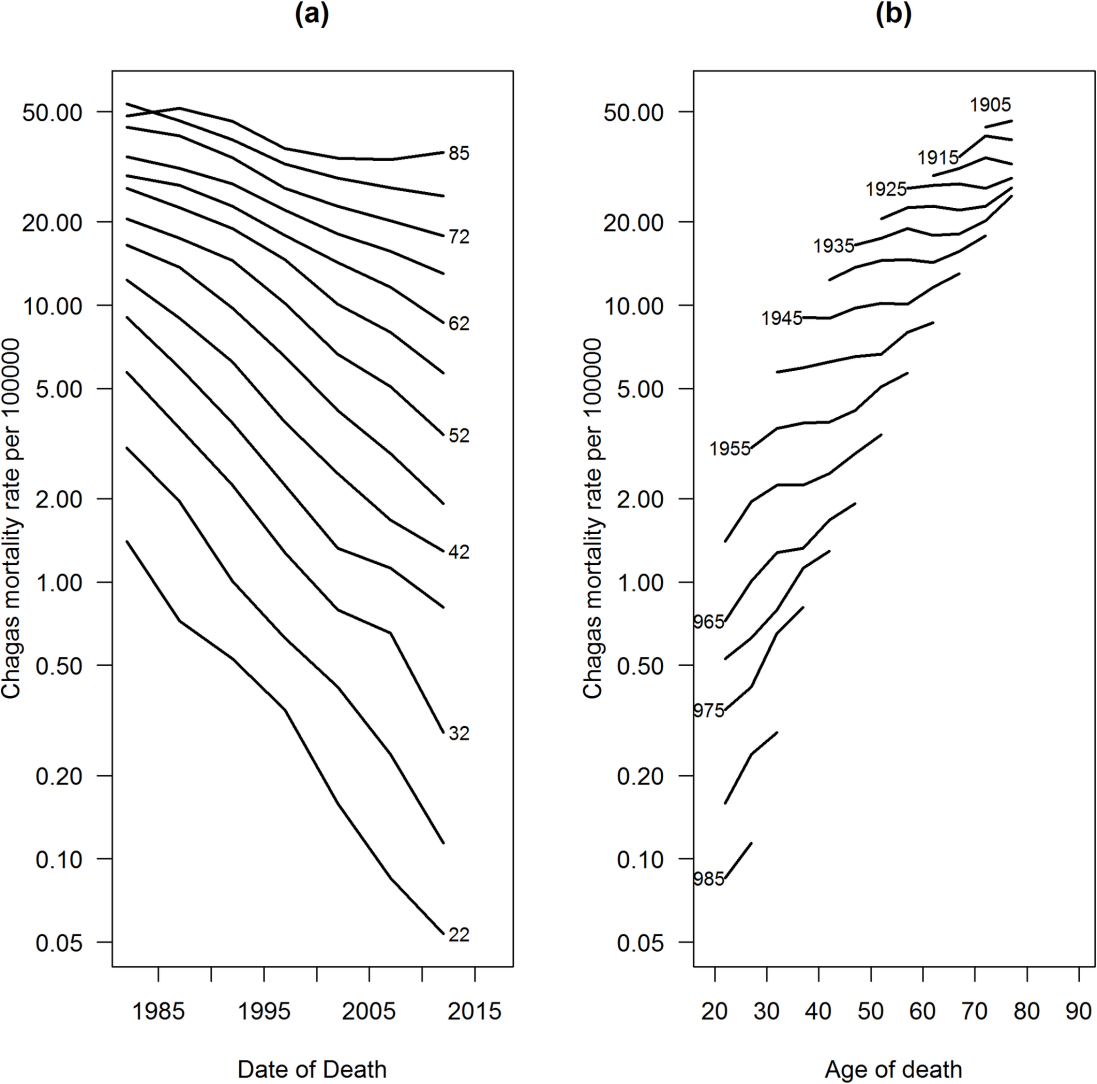
Distribution of standardized mortality rates due to Chagas disease in Brazil from 1980–2014. Period-specific rates according to age (a) and age-specific rates according to birth cohort (b).

The averages of five-year standardized mortality rates for the whole country increased. The highest rates occurred in the 1980s, and the lowest rates occurred in the 2000s. The lowest average rate occurred in the last period analyzed (2010–2014) (Table 2). Descriptive analysis of the temporal effects (age, period and birth cohort) showed that mortality rates increased progressively with age, where the highest rates occurred in the age group of 80 years and over. Mortality reduction was also observed in all age groups, since the 1990s. The highest mortality rates occurred in individuals born in the 1900–1905 cohort, and the lowest mortality rates occurred in the 1990–1994 generation (Fig 1A and 1B).

**Table 2:** Crude and standardized, as well as uncorrected and corrected, mortality rates for Chagas disease in Brazil per 100,000 inhabitants by geographic region and sex from 1980 to 2014.

				Period					
Subgroup	Rates	1980–84	1985–89	1990–94	1995–99	2000–04	2005–09	2010–14	Total
	UCMR^1^	6,15	5,36	4,52	2,15	2,72	2,58	2,40	2,70
	USMR^2^	7,66	6,54	5,31	2,59	3,17	2,85	2,41	3,85
Brazil	CCMR^3^	9,48	8,13	6,69	5,48	4,01	3,33	5,48	4,28
	CSMR^4^	12,87	10,88	8,67	6,83	5,40	3,98	3,22	4,85
	UCMR^1^	2,50	2,09	1,76	1,46	1,18	0,97	0,81	1,44
	USMR^2^	3,24	3,14	2,49	2,25	1,71	1,18	0,90	1,61
South	CCMR^3^	3,00	2,44	1,98	1,60	1,27	1,03	0,85	1,61
	CSMR^4^	3,88	3,67	2,81	2,47	1,83	1,25	1,25	1,80
	UCMR^1^	6,27	5,27	4,39	3,55	2,79	2,32	1,96	3,48
	USMR^2^	9,91	8,29	6,79	5,43	4,12	3,13	2,51	3,26
Southeast	CCMR^3^	7,19	5,94	4,99	4,04	3,13	2,55	2,11	3,91
	CSMR^4^	11,32	9,33	7,72	6,16	4,63	3,44	2,70	4,17
	UCMR^1^	0,33	0,49	0,59	0,50	0,47	0,47	0,55	0,50
	USMR^2^	0,50	0,79	0,98	0,96	0,88	0,79	0,84	0,84
North	CCMR^3^	0,48	0,74	0,90	0,72	0,64	0,57	0,67	0,66
	CSMR^4^	0,72	1,19	1,51	1,39	1,20	0,95	0,96	1,12
	UCMR^1^	1,78	1,88	1,75	1,74	1,73	1,93	1,95	1,83
	USMR^2^	2,37	3,00	2,27	2,43	2,28	2,34	2,09	2,33
Northeast	CCMR^3^	3,33	3,45	3,00	2,66	2,42	2,17	2,13	2,64
	CSMR^4^	4,47	4,61	4,72	4,46	3,18	2,62	2,28	3,33
	UCMR^1^	26,50	21,65	16,54	10,48	9,49	7,45	12,52	11,97
	USMR^2^	40,42	32,00	23,23	18,15	15,07	12,13	8,92	16,91
Central West	CCMR^3^	35,58	28,33	20,00	12,37	10,37	7,87	7,51	14,24
	CSMR^4^	54,45	41,95	28,14	21,43	16,49	10,54	9,28	20,02
	UCMR^1^	7,22	6,33	5,34	1,77	3,37	3,00	2,70	3,75
	USMR^2^	9,31	7,99	6,48	9,31	4,21	9,31	2,71	4,01
Men	CCMR^3^	9,48	8,13	6,69	5,48	4,01	3,33	2,93	5,25
	CSMR	12,21	12,62	10,10	7,96	6,24	4,60	3,68	6,08
	UCMR^1^	4,93	4,29	3,63	2,46	2,14	2,17	2,10	2,81
	USMR^2^	5,96	5,08	4,13	3,05	2,40	2,41	2,11	3,18
Women	CCMR^3^	6,25	5,35	4,49	2,98	4,48	2,37	2,25	3,34
	CSMR^4^	7,59	6,37	5,14	3,70	4,16	2,63	2,25	4,32

^1^UCMR Uncorrected Crude Mortality Rate.

^2^USMR Uncorrected Standardized Mortality Rate.

^3^CCMR Corrected Crude Mortality Rate. Mortality rate after the correction for deaths due to ill-defined cause, with age and sex ignored.

^4^CSMR Corrected Standardized Mortality Rate. Standardized mortality rate after the correction for deaths with ill-defined cause, with age and sex ignored.

**Fig 2:**
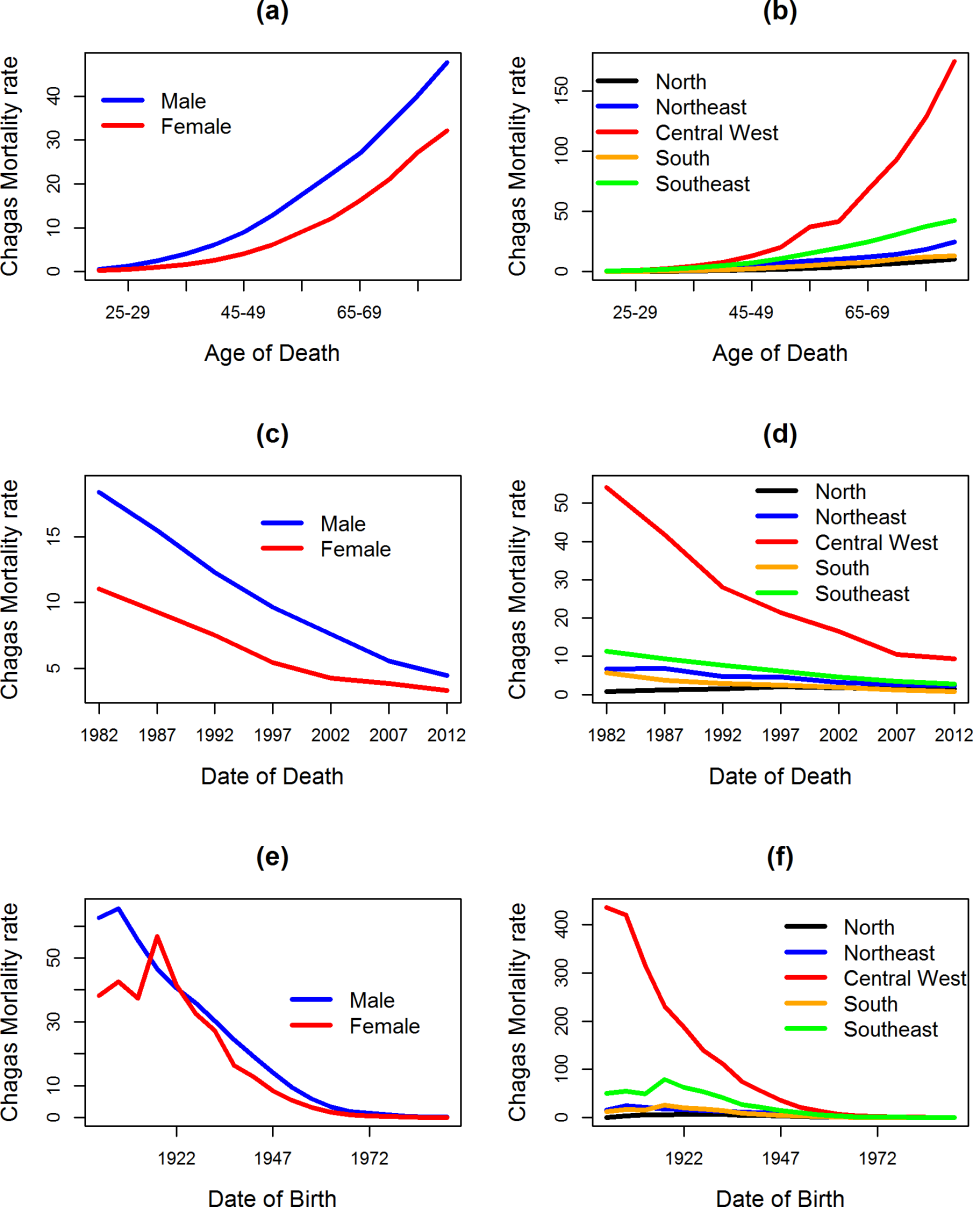
Distribution of the observed average mortality rates for Chagas disease according to sex and geographic region in Brazil from 1980–2014. Standardized rates of Chagas disease mortality according to the observed effects of age (a,b), period (c,d), and birth cohort (e,f) by sex (first column) and geographic region (second column).

The highest mortality rates were observed for males, with a rate of 6.08 deaths/100,000 males in the period, a rate 1.61 times higher than the female rate (4.32 deaths/100,000 females). Regarding the effects of age, period and cohort, mortality rates by sex were higher, on average, among men for all temporal effects, except in the generation of the 1920s, when the mortality rate in women was higher. For women, the rate was 56.82 (1920–1924) and 41.39 (1925–1929), while for men it was 46.61 (1920–1924) and 40.61 (1925–1929) deaths/100,000 individuals (Fig 2A, 2C and 2E).

The observed temporal evolution of the rates in the geographic regions, according to the effects of age, period and cohort, showed a heterogeneous pattern. The highest average rate occurred in the Central West region (20.02 deaths/100,000 inhabitants), and the lowest rate occurred in the North region (1.12 deaths/100,000 inhabitants) (Table 2). Among the regions, the pattern of temporal effects was similar to Brazil as a whole, except in the North region, which had the lowest rate from 1980–1984 (Table 2). There was also a -75.0% reduction when comparing the rates of the first period (12.87 deaths/100 thousand inhabitants) and the last period (3.22 deaths/100,000 inhabitants) of the historical series in Brazil: -83.0% (Central West), -67.8% (South), -76.1% (Southeast) and -49.0% (Northeast). This was in contrast to an increase of +33.3% in the Northern region (Table 2). There was a significant reduction in mortality rates for birth cohorts from the 1960s onwards in all regions (Fig 2B, 2D and 2F).

### Modeling results

**Fig 3:**
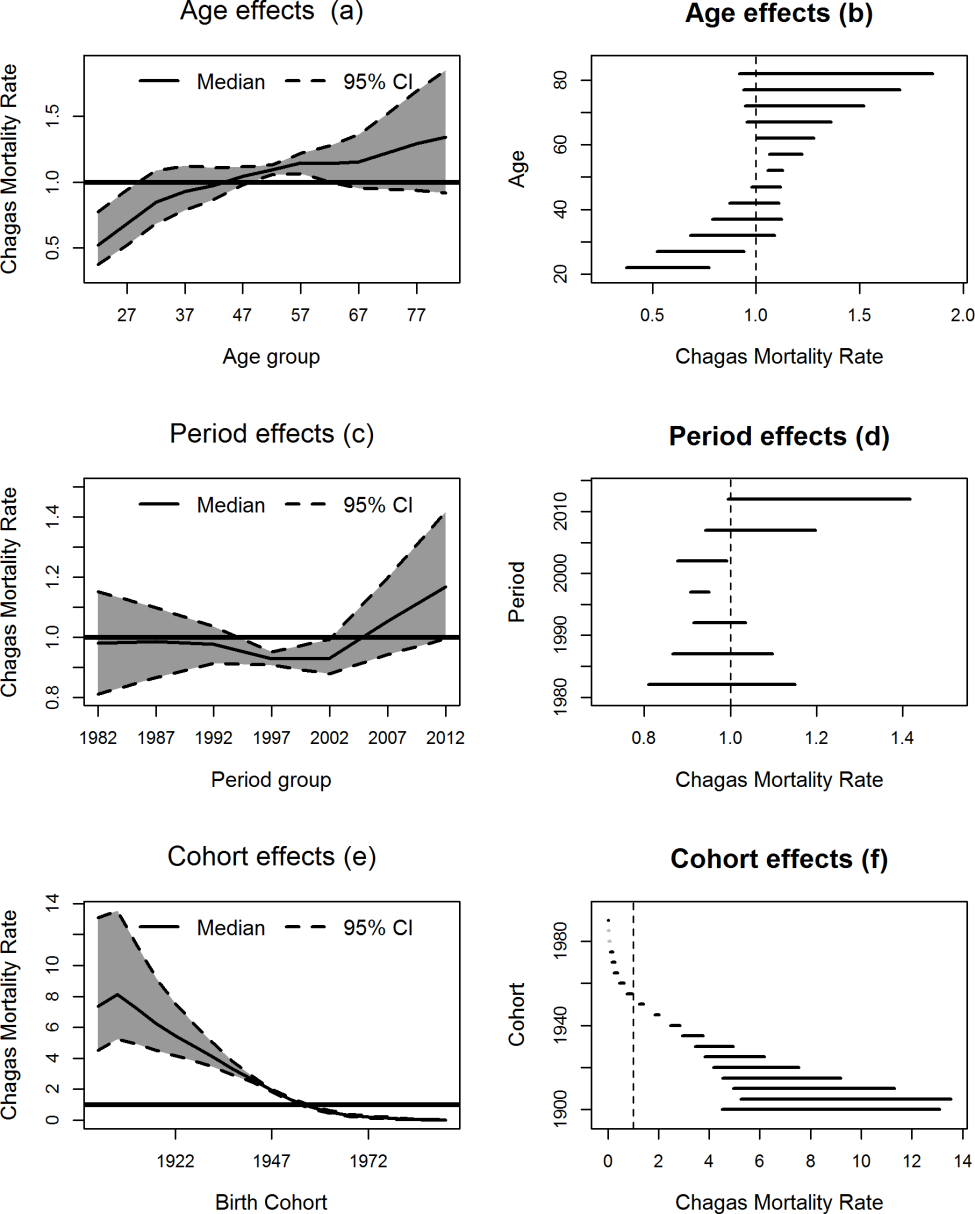
Estimated rates of death by Chagas disease according to temporal effects. Estimated mortality rates from Chagas disease and credibility intervals (95%) for the effects of age (a, b), period (c, d), and birth cohort (e, f) in Brazil from 1980–2014.

The univariate APC model estimated in Brazil showed that the age-specific death risk for CD ranged from 0.37 to 1.85, and it showed a tendency of increasing with age. People from 20 to 29 years of age had the lowest rate (Estimated 0 < RRR < 1), and people from 50 and 64 years of age had the highest rate (Estimated RRR > 1) (Fig 3A and 3B). The period-specific death rates ranged from 0.81 to 1.42, with the lowest rates in the periods from 1995 to 1999 and from 2000 to 2004. In the more recent periods, although it was not significant, there was an increasing trend in mortality rates (Fig 3C and 3D). The cohort-specific rates ranged from 0.01 to 13.6. The rates were higher among older cohorts and 0 < RRR < 1 for people born after 1955 (Fig 3E and 3F). The random effects that incorporated the overdispersion (z's) of the data were significant, with rates varying between 0.86 and 1.17.

**Fig 4:**
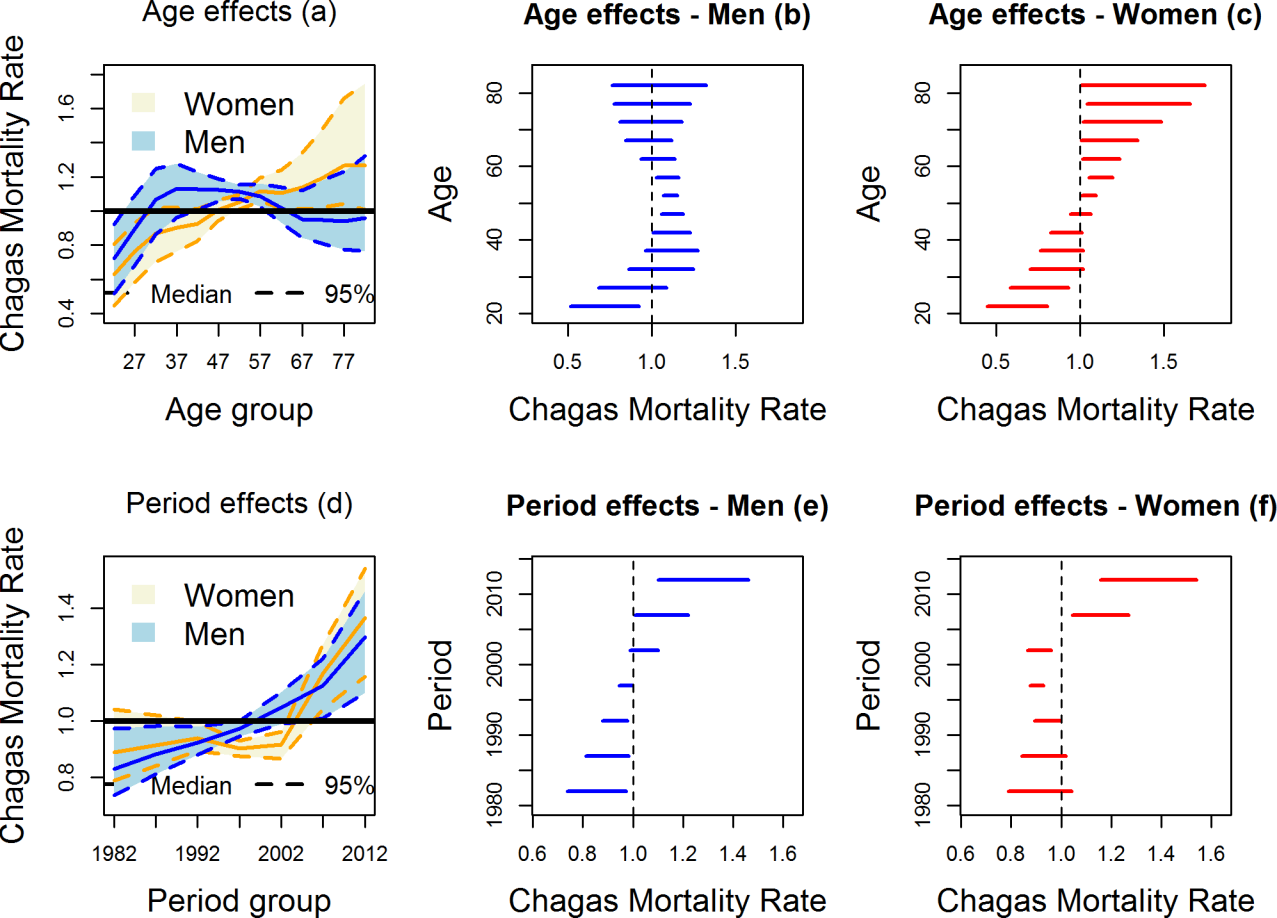
Estimated rates of mortality due to Chagas disease according to sex. Estimated mortality rates for Chagas disease and credibility intervals (95%) according to sex, for the temporal effects of age (a, b, c) and period (c, d, e) in Brazil from 1980–2014.

Based on the Table 3 results, among the multivariate BAPC models considering sex (models ·a), the best fitted model was the model with age effect and period effect specifics by sex (model 5a). The results showed that women had lower death rates for 5 years more than men. For males, 0 < RRR < 1 until the age of 24, and for females, 0 < RRR < 1 until the age of 29. In addition, men had high death rates from 40 to 59 years, and women from 50 years of age had increasing death rates (Fig 4A, 4B and 4C). The death rates for both sexes were high and significant after 2005; however, even when 0 < RRR < 1, the rates were higher among men from 1995 to 2004 (Fig 4D, 4E and 4F).

**Table 3:** Comparison of fitted models with temporal terms, overdispersion random effects, and heterogeneity by sex and geographical regions (Prioris RW1 for temporal effects).

Fitted models considering heterogeneity by sex **(Chosen model: 5a)**							
Models	1a	2a	3a	4a	5a	6a	7a
Log Score	6.032	5.716	6.033	5.864	5.594	5.795	5.704
DIC	349.76	324.457	349.858	334.636	318.462	330.890	332.539
Fitted models considering heterogeneity by geographic regions **(Chosen model: 7b)**							
Modelo	1b	2b	3b	4b	5b	6b	7b
Log Score	5.334	5.251	5.171	5.074	4.836	4.730	4.630
DIC	920.728	928.478	877.157	922.776	847.647	809.50	755.13

**Fig 5:**
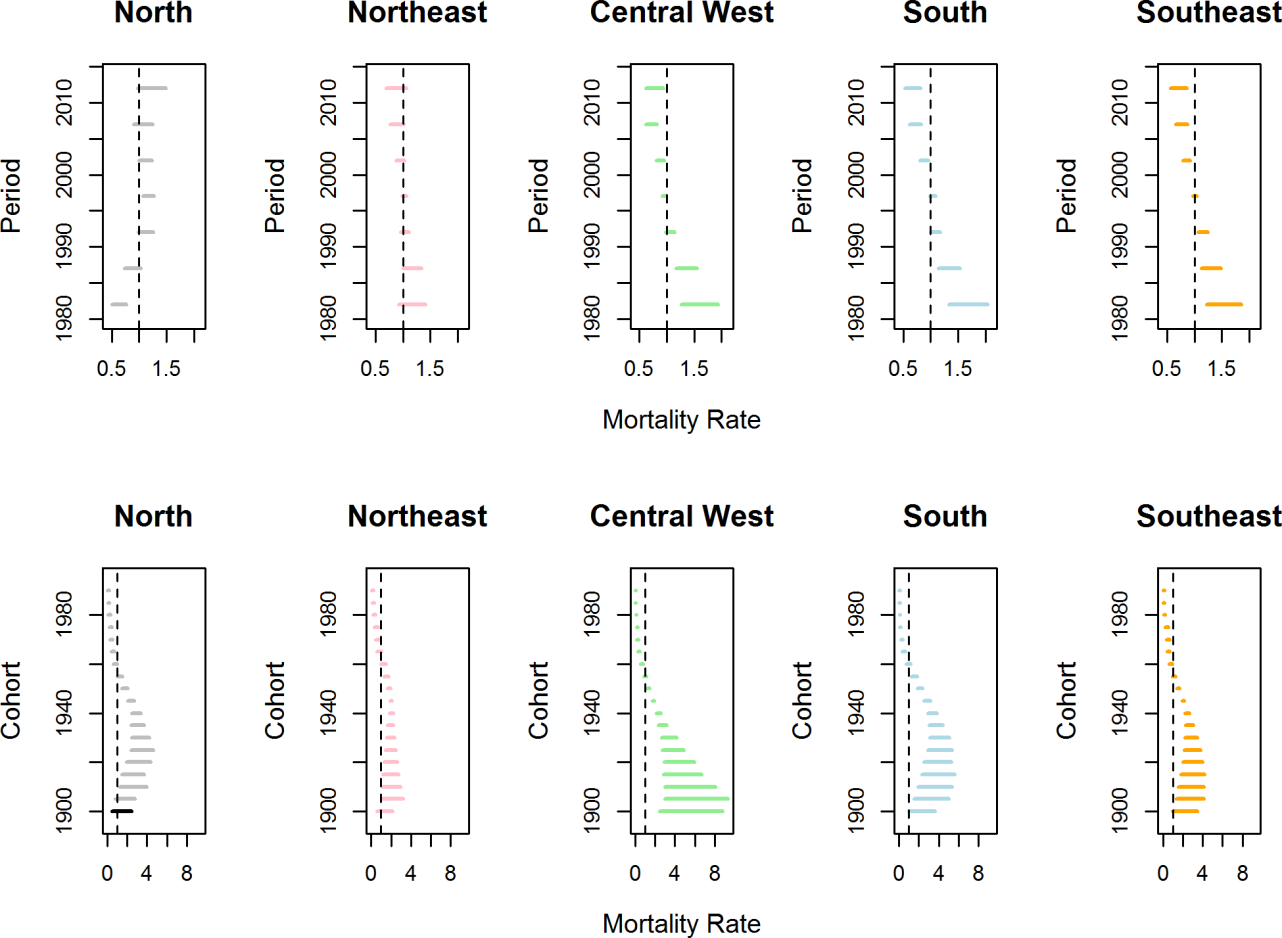
Estimated mortality rates from Chagas disease according to geographic region. Estimated rates of mortality due to Chagas disease and intervals of credibility (95%) according to the effects of period (first line) and birth cohort (second line) for different Brazilian regions from 1980–2014.

For the Brazilian regions (models ·b), the best fitted model considered period effect and cohort effect specifics by region. Fig 5 shows changes in the death rates through the regions, according to periods (first line) and birth cohorts (second line). During the study period, there was a reduction in the death rates in all regions, with the exception of the North region. The North had a low rate until 1985–1989, followed by a subsequent increase, but rate changes were only significant from 1995 to 1999. The South, Southeast and Central West regions had similar profiles. The rates in these regions were RRR > 1 and were significant until 1990–1994, with a significant reduction in the three quinquennia of the years 2000 (2000 to 2014). There were no significant rate changes according to periods for the Northeast region. The death rates due CD were significant and were higher than 1 for cohorts born until 1955 for the Central West and Southeast regions and until 1960 for the other regions. For younger cohorts, 0 < RRR < 1 (Fig 5).

### Predictions

**Fig 6:**
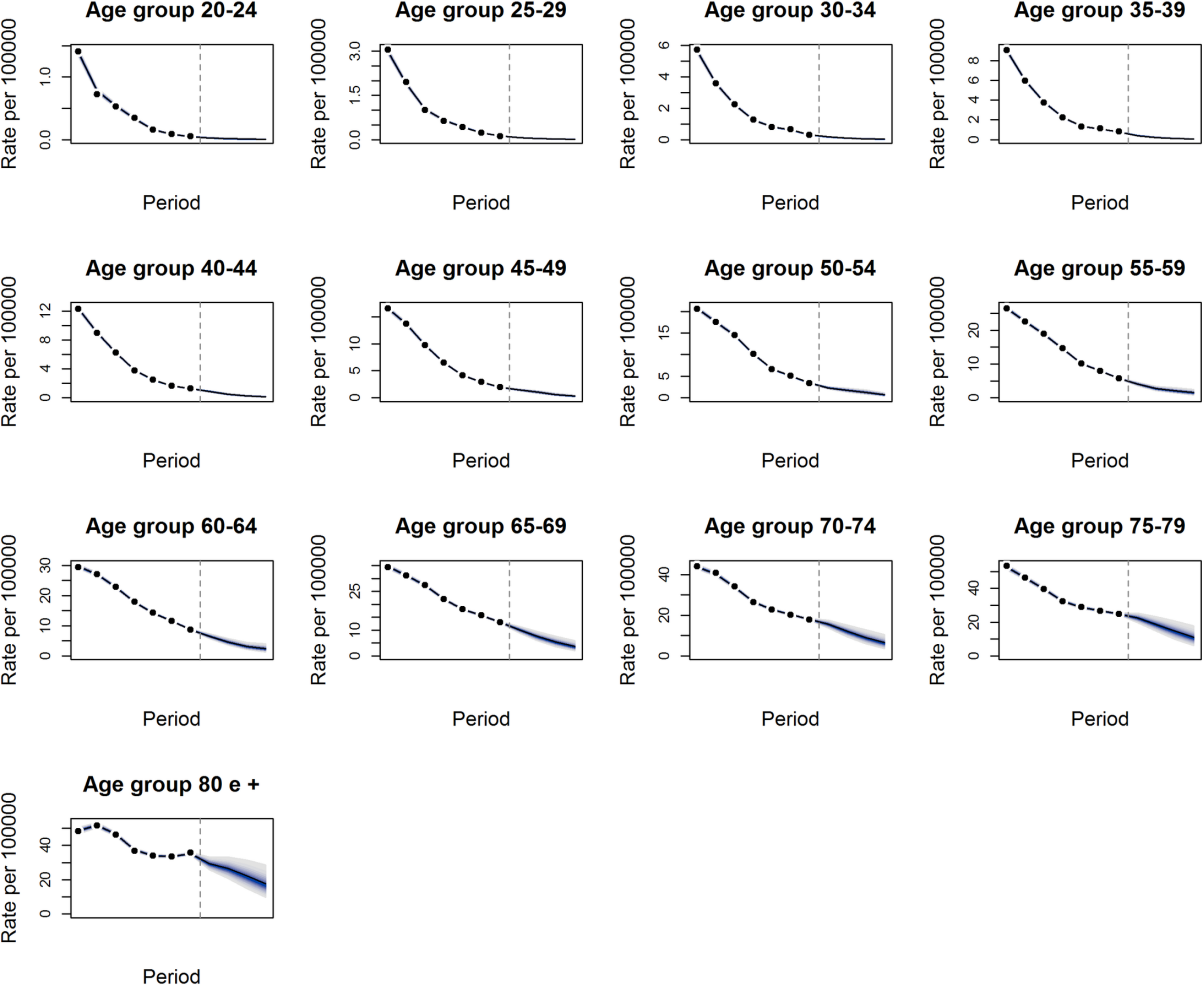
Predicted mortality rates for Chagas disease from 2015–2034. Adjusted age-specific rates for Chagas disease mortality and confidence intervals (95%) according to age groups, projected from 2030–2034 (predictions from the vertical line) in Brazil.

The predictions of Chagas death rates in Brazil for the next 20 years (2015–2034) indicate a progressive reduction in death rates for all age groups, with a higher reduction in younger age groups that progressively decreases with age (Fig 6). For the 20–24 age group, the estimated rate was 0.0490 deaths/100,000 inhabitants in 2010–2014 and 0.007 deaths/100,000 inhabitants in 2030–2034, representing a reduction of 86% over twenty years. For the age 80 years and older age group, the estimated rate in 2010–2014 was 35.146 deaths/100,000 inhabitants, and the rate was17.612 deaths/100,000 inhabitants in 2030–2034, representing a reduction of only 50% in the same period.

## Discussion

Although many social transformations have occurred over the years in endemic and non-endemic regions for Chagas disease (CD), it continues to be an important problem for public health in Brazil with new challenges and epidemiological scenarios such as oral transmission and decentralized health systems. In addition, there are difficulties regarding the production and distribution of therapeutic drugs, specialized medical knowledge, and appropriate access for the diagnosis and treatment of disease [43]. The temporal effects on CD mortality varied by sex and region in this study. The data analyzed showed a progressive increase in the risk of CD death with increasing age, up to 30 years of age. The highest mortality rate occurred between 50 and 64 years of age. There was a generational effect, with a lower risk of death for younger cohorts. There was a reduction in the risk of CD death for the Central West, South and Southeast regions. There was no statistically significant change during any period in the Northeast region, but there was an increased risk in the North from the year 2000. There is a projected reduction in mortality rates for the next 20 years, which will be highest among the youngest patients.

The coverage quality of information systems has an important role in health surveillance, especially in monitoring mortality. However, the information quality of death registries in Brazil, in addition to regional disparities in access to health services, can lead to underestimated mortality rates, even with improved coverage over the years [28],[29],[44]. Thus, in this study, we corrected the number of deaths due to CD according to the records of ill-defined death causes while, ignoring age and sex, resulting in a 27% increase in CD as a basic cause of death. There was verified that there was no change in the temporal evolution of mortality during the seven quinquennia analyzed; nevertheless, it allowed us to work with more reliable records of death.

We verified that the most important factor associated with the temporal evolution of Chagas mortality in Brazil was the birth cohort, which is generally not considered in temporal trend studies. This evidence suggests that, in the last three decades, the deaths reported due to CD mainly occurred in older generations. These people were more commonly exposed to the main vector responsible for transmission and were infected at a time when therapeutic drugs and diagnoses were less advanced.

The CD death rate was low (0 < RRR < 1) for people born after 1960. The lower mortality after the 1960s may also be a consequence of the migration process that occurred in Brazil, especially in the 1950s and 1960s, when large population groups moved from rural areas with a high risk of infection to urban areas [45]. Martins-Melo et al. (2014) attributed the mortality reduction and the increase in the survival of infected individuals in younger cohorts to a better knowledge of the natural history of the disease and to the clinical and surgical care improvements [14].

These study results also reinforce the success of the National Chagas Disease Control Program. After the control measures were implemented, the younger generations were less exposed to infection by this parasite. Thus, a high prevalence of infection by this parasite in older people is expected due to the blockade of infection in the younger cohorts who live in areas that were previously endemic [12],[18],[21],[46]. In addition, the increase in the elderly living with CD can be explained by the reduction in the severity of the clinical aspect of the disease, caused by the interruption of reinfection, and the expansion of access to health services with the implementation of the Unified Health System (SUS) in the country [20],[43],[46],[47].

We showed that the death risk was lower between 1995 and 2004, possibly as a result of the decrease in classical vector transmission in endemic areas from 1990 onward, when intergovernmental programs were proposed in order to combat the proliferation of the disease [43]. Nóbrega et al. (2014), who analyzed the mortality rate by CD, second year of occurrence, sex and age in Brazil from 2000 to 2010, also observed a drop in the standardized mortality rate. The rate decreased 32.4% during the period [10]. Also as the CD control actions results, The *T*. *cruzi* infection prevalence in the adult population was also reduced, ranging from 4.2% (6.5 million) in 1975–1980 to 1.3% (1.96 million) in 1995 [48]. Another study carried out in 1994 at an endemic area in Minas Gerais state (Bambuí) showed a seroprevalence of zero at up to 19 years of age; and of 37.7% in the age group above 60 years [21], indicating transmission interruption in recent years. A study of the seroprevalence in children under 5 years of age showed vector transmission in only 0.01% of children [49].

In contrast to previous studies, we observed a trend of increasing mortality due to CD from 2005 onward, although the trend was not statistically significant. This shows that there was no the expected decline in the death risk after the discontinuation of *Triatoma infestans* in 2006, and that, on the other hand, we know that the increase in the mortality rate may be associated with the predominance of cases in the acute phase in more recent years. However, on average, we have predicted a progressive decline in Chagas mortality for the next two decades in the country.

It was observed that the CD mortality rate increased with increasing age up to 30 years and that the death risk was higher for people aged 50–64 years. Some studies, particularly population-based studies in cohorts with long follow-up periods, point to the aging pattern of the Chagas population and to the increase in ages in the higher age groups [50],[51].

We found that in all periods, the mortality rates were higher in males than females, which is similar to other studies [10],[12],[15],[17],[20]. These authors explain that the result is due to women accessing more health services for primary and secondary prevention actions than men, who are inhibited by socio-cultural issues associated with the construction of masculinity. Consequently, many men may be diagnosed in advanced stages of the chronic phase of disease, reducing their survival in relation to women.

We also verified that the temporal effects of age and period varied according to gender. Men had a higher death risk between the ages of 40 and 59 years, and women had a higher death risk at age 50 and older. In addition, the risk for both sexes was high and significant after 2005, although it was higher among men in the period from 1995 to 2004. Nóbrega et al. (2014) observed that the majority of deaths by CD (85%) occurred in men over 60 years of age, and the main cause of death was cardiac involvement [10]. In addition, considering multiple causes of death by CD, a study conducted in São Paulo between 1985 and 2006, showed a mortality coefficient drop of 51.34%, which was lower among women, with a shift of deaths to more advanced ages [20].

The highest mortality rates were observed in the Central West and Southeast regions. This depicts T. cruzi infection in the past decades because the mortality rate reflects a large proportion of patients with chronic Chagas disease [10],[12],[15],[17],[20]. Regarding temporal evolution, period and cohort effects differed between regions. The death risk remained null and stable in the Northeast region throughout the period. Although not significant, there was a trend of increased death risk in the North region that may represent a significant problem over the next years. For the other regions, there was a decrease in risk over time. The risk was significant and more than 1 for cohorts born until 1955 in the Central West and Southeast and until 1960 for the other regions.

Drummond and Marcopito (2006) found that the mortality coefficient between 1981 and 1998 declined in the Southeast, South and Central West regions, which was different from the North and Northeast [19]. Nóbrega et al. (2014) observed a decrease in mortality due to cardiac involvement among most Brazilian regions, except in the North. However, the number of deaths due to digestive impairment increased in all regions [10]. On the other hand, Martins-Melo et al. (2012b) showed a significant increase in mortality in the North and Northeast regions and a reduction in the other regions [15]. The stability or trend of growth in these mortality rates is worrisome due to the constant measures of control of the disease in the country. This suggests, the need for different measures and policies in these regions and strategies directed at oral transmission in the North region.

The North and Northeast regions have been considered areas of low incidence; however, several studies have shown a trend of ascending mortality by CD in these areas [12],[17],[19]. It is presumed that the complexity of controlling triatomines is of secondary importance (*T*. *brasiliensis* and *T*. *pseudomaculata*) to the congenital and oral transmission of *T*. *cruzi*, which have contributed to the maintenance of transmission in the Northeast region of Brazil [15],[52–57].

In the Northern region, it is believed that the increased mortality trend is due to the improvement in the coverage and quality of death records, which is associated with increased surveillance due to the increase in oral transmission in the 2000s. In addition, the migratory flow to this region in the 1970s and 1980s played an important role in CD deaths, since more than 80% of deaths in this region between 1981 and 1998 occurred in persons born in other states of the country [10],[19].

The Amazon region, which corresponds to most of the Northern region of Brazil, was not considered a priority area for CD prevention and control since it had a low prevalence of the disease and low morbimortality [4],[58]. However, there is no doubt that the transmission of this protozoan is a reality in this region. In the 2000s, there was an increase in the number of acute cases, especially in the state of Pará [9]. From 1999 to 2007, there was an increase of more than 20% in acute cases in Brazil, with 11% occurring in the North region [12].

We included age-specific rates in one set of rates for each stratum, sex and geographical region that could be analyzed separately by means of a classical APC model. However, because of similar relevant risk factors, it was beneficial to analyze all sets of rates jointly, treating some sets of time effects as common across strata. Multivariate APC models share sets of time effects, while the remaining parameters can be different. The differences in stratum-specific time effects in multivariate APC models are identifiable and can be interpreted as log relative risks so that the identifiability problem for univariate APC models is avoided [35].

Counterpoints to this work are the information quality and the mortality information system coverage. In the present study, we performed death records correction for ill-defined causes while ignoring sex and age, but we did not correct the underreporting of deaths, and therefore, the Chagas disease mortality rates may be underestimated. In addition to that, the available death information does not discriminate between the acute and chronic phases of the disease, which have had different predominance over time in Brazil. Epidemiological surveillance focuses on the acute phase of the disease, which is the unique phase of compulsory notification in the country, so the mortality rates are always underestimated. We also have not considered the covariables sex and residence region in the predicted rates. In future works, we should consider inequalities due to race and smaller spatial units, such as federated units and municipalities. Additionally, the quality of the fitted models should be retrospectively assessed by cross-validation methods.

Our findings are in line with the reduction of exposure to *T*.*cruzi* infection, the improvement of living conditions and the health of the population, and the migration process from rural areas to urban areas. We believe that the findings on the cohort effects for CD mortality are a result of the period effect promoted by the Disease Control Program, the expansion of access to health care with the implementation of SUS, and changes in Brazilian population dynamics (migration between 1950–1960), as well as improvements in socioeconomic conditions and reduced population exposure to T. cruzi infection. In addition, the differences among the regions of the country reflect the association of mortality due to the disease with socioeconomic status, as well as inequities in access to healthcare in the Brazilian population.

## Supporting information

S1 Dataset (ZIP)
